# The work-related stress experienced by registered nurses at municipal aged care facilities during the COVID-19 pandemic: a qualitative interview study

**DOI:** 10.1186/s12912-022-01059-x

**Published:** 2022-11-02

**Authors:** Cilmara Arén, Armand Jaçelli, Berit Gesar, Ingrid From

**Affiliations:** School of Health and Welfare, Dalarna University, Falun, Sweden

**Keywords:** Experiences, Nursing care, Older people, Registered nurse, Work-related stress

## Abstract

**Background:**

Stress can originate from many different unsatisfying work situations. Registered nurses working in municipal care have experience of work-related stress in different ways.

**Aim:**

The purpose of this study was to describe the work-related stress experienced by registered nurses caring for older people at municipal aged care facilities.

**Methods:**

Qualitative semi-structured interviews according to Polit and Beck were carried out in clinical work at six different municipal aged care facilities in Sweden. Twelve registered nurses participated in the study.

**Results:**

The results outlined in one main central theme: *Feelings of inadequacy and dissatisfaction contribute to work-related stress* and three categories: *Difficulty coping with work tasks, Insufficient support, Work-related stress affects private lives.* Areas identified were lack of time, staff shortages, high number of patients, lack of communication and teamwork in the working group, showing that inadequacy and dissatisfaction can contribute to work-related stress. This can contribute to work-related stress, and it can be a result of problems in the organizational and social work environment.

**Conclusion:**

This study showed the everyday experiences of registered nurses’ stress at work. The reasons that registered nurses experience a heavy workload were found to be similar in several municipal care facilities. Future interventions should consider the areas of stress found in this study to reduce the risk of further increasing the work-related stress experienced by registered nurses working in municipal aged care.

## Background

The World Health Organization, WHO (2017) has classified stress as the “health epidemic of the 21st century” and stated that work-related stress is the reaction that people may have when presented with work demands and pressures beyond their experience and capabilities. Personal stress arises in a wide range of work situations when employees feel they haveinsufficient support from managers and colleagues and inadequate control over work processes [1]. Stressors of emotional or physical tension are described by Yao et al. [2] as any event or thought that trigger people to feel frustrated, angry, or nervous.

Previous research points out that registered nurses working in municipal care experience different work-related stressors, impacting their physical and mental well-being [3, 4]. Understaffing and the lack of good relationships at work between colleagues, such as different views and experience, can negatively affect mental health [3, 5]. Registered nurses’ health in older people’s care affects the quality of patient care and safety. Working under severe stress is one of the reasons for absence from work [6]. Additionally, Fang et al. [7] describe how registered nurses who feel a significant commitment to their work can become too committed, leading to increased work-related stress. Work-related stress is the most important contributor to poor work satisfaction [7]. According to Hassan et al. [8] and van Steijn et al. [9], work-related stress contributes to work-related diseases as anxiety and depression.

The setup of the organization and lack of support from management both in emergency care [6] and care of older people in a local municipality complex and may cause stress [10]. Work-related stress is an increasing problem associated with dysfunctional workplaces [11]. Anshasi et al. [12] described the fact that there is a significant need for competence development in older people’s care among nursing staff in municipal care facilities. Organizations must improve the work situation to increase an employee’s health-related quality of life without stress. Programs to improve quality of life without stress should focus on promoting continuous education for their employees, positive relationships between colleagues, social support, and stress-reduction courses [13].

Work-related stress among registered nurses raises significant concerns about patient safety, nurses’ attitudes toward their patients in nursing homes and aged care facilities, and the quality of aged care [14, 15]. The internal stressors can contribute to the work sometimes being carelessly done, leading to an increased risk of making serious mistakes [16]. People perceive and handle stressful situations in different ways because humans are different and experience things differently [16]. Bittinger et al. [17] describe work-related stress as dangerous and harmful for registered nurses’ work situation. Counteracting this may be the key to reduce the adverse effects working in a stressful situation with the risk of reducing quality of life.

Furthermore, studies have shown that a long period with a heavy workload can cause many other negative impacts, such as critical incidents or high staff turnover among registered nurses [14, 15]. According to Antonovsky [18] the experience of feeling connected, the Sense of Coherence, determines what degree of mental health the individual can feel. In the demand-control-support model by Karasek and Theorell (1990), they describe the relationship between health and working life stress. This model shows which conditions in the work environment can decrease stress. The main concepts are demand, control, and social support. The authors emphasize the importance of a social network in reducing work-related stress and how it can help to reduce the risk of illness in the workplace [19].

Staff shortages and understaffing in nursing are problematic and can negatively affect the quality of care. Nurses should have satisfactory working conditions and therefore it is essential to identify sources of stress affecting registered nurses [20]. People are suffering from work-related stress conditions and there is a need to be more proactive in reducing work-related stress [1].

Work-related stress for a registered nurse has proven to be a danger to patient safety and quality of care as well as to the nurse’s own physical or mental well-being and it can lead to them becoming overworked and a decreased quality of life. To understand work-related stress conditions, it is essential to study nurses’ experiences of work-related stress. According to our knowledge, few studies have focused on stress among nurses in municipal care.

### Aim

The purpose of this study was to describe the registered nurses’ experiences of work-related stress in the care of older people at municipal aged care facilities.

## Method

### Design

This study was an empirical qualitative study based on semi-structured interviews [21]. The data was analyzed with qualitative content analysis according to the description by Graneheim and Lundman [22]. Qualitative content analysis can help and show conflicting opinions or unsolved issues regarding the meaning and use of concepts, procedures, and interpretation. Using this method provides an important overview of the main concepts related to qualitative content analysis [22].

### Sample selection

The selection of participants was made using convenience sampling because the data was collected in six different municipal aged care facilities in Sweden [21]. Inclusion criteria for participation in the study were that registered nurses should have worked for at least one year or more in an aged care facility. No criteria for exclusion were applied. All participants were registered nurses who had between 1–40 years’ nursing experience which included the experience of working in aged care at a municipal care facility. Twelve registered nurses participated in the study. Seven of the participants were women and five were men. They were all aged between 25–70. Seven had advanced nurse practitioner qualifications and five had a registered nurse qualification. The selected sample is representative of this group characteristic according to gender and age distribution and experiences. The aim was to obtain a good heterogeneity, and this was easily achieved due to the multiple diversity of the gender, age, and experience of our participants in the conducted interviews. Also, to maximize the variety of the participants and respond to the relevant research question. Before starting the interviews’, written information was provided, and a written consent form was signed by the manager of the respective facility. After consideration, the interested registered nurses contacted the authors and accepted participation by signing and returning a letter of consent.

### Data collection

The interviews were carried out at the participant’s workplace. All the participants in the study were informed that they could discontinue the participation at any time without stating the reason. According to Polit & Beck [21] a semi-structured interview is an interview based on several open questions of the phenomenon being investigated. Eight open questions were chosen to be relevant to the study with one or more follow-up questions on the same question to gain a deeper insight into the area of interest studied. The questions were about stress situations at work and covered their daily work in general but also more detailed discussions of when and why stressful situations occurred and how they handled them. The interview included guide the following questions: ‘Describe your working experiences as a nurse in a municipal care for old people’, ‘describe what work-related stress means for you´ and ‘does the work-related stress affect your quality of life? Describe how’. All interviews were conducted in Sweden, in March 2021. Informed consent was obtained, both orally and in writing, before the start of the interview. One of the authors (CA) was responsible for booking interview times with the participants. They also carried out all the individual interviews. The data collection was done through the help of an interview guide according to [21]. All the interviews took place in private rooms and non-disturb sign was placed outside the door to minimize the risk of errors during the interviews. The individual interviews lasted about 4045 minutes and were recorded using a digital voice recorder and transcribed verbatim.

### Data analysis

The qualitative content analysis method used was according to Graneheim and Lundman [22]. The interviews were listened to several times by two authors (CA, AJ) independently and transcribed into text. The transcribed text content was divided into areas based on the research questions. Parts of the content related to the aim were set as meaning units that were then condensed and coded. See an overview in Table 1. The codes were compared to identify and describe variations and similarities in the textual content to answer the aim of the study. Parts of the text content that responded to the aim were removed and set as meaning units that were then condensed and coded in the end. The codes were compared to identify and describe variations and similarities in the textual content to answer the study’s aim, as was described by Graneheim and Lundman [22]. To ensure the credibility of the codes, they would be checked against condensations and meaning units. Then the codes would be sorted into categories based on similar content, and these categories would be analyzed to answer the aim of this study. As described by Polit and Beck [21], a transcript must be accurate and fully reflect the content of the interview. The data were analyzed through a qualitative content analysis at both manifest and latent levels, according to Graneheim and Lundman [22].

**Table 1:** Examples of meaning units, condensed meaning units and codes

Meaning units	Condensed meaning	Codes
Then you can get physical problems such as, gastritis or palpitations or that you sweat or something and you feel stressed.	Stress can cause various physical problems such as gastritis, palpitations, or sweating.	Stress can cause physical problems.
There is also a stress and pressure, and you have patient safety and must prioritize as the managers say prioritize, prioritize and it is difficult and what should we prioritize when we have our system that the municipality has established.	Stress and pressure, patient safety, managers say prioritize, but the municipality has established a system.	Stress due to prioritization during low staffing periods.

This study was approved by the ethics committee at Dalarna University: HDa dnr 7.1.1 2021 /159.

## Results

The result describes the registered nurses’ experiences of work-related stress. All interviews were conducted in Sweden, in March 2021.The analysis resulted in one central theme *“Feelings of inadequacy and dissatisfaction contribute to work-related stress”* three categories and ten subcategories that responded to the study’s purpose. See an overview in Table 2.

**Table 2:** Overview of central theme, categories, and subcategories

*Theme*	Feelings of Inadequacy and dissatisfaction contribute to work-related stress									
***Categories***	**Difficulty coping with work tasks**					**Insufficient support**			**Work-related stress affects private lives**	
***Subcategories***	Lack of time	Understaffing	Lack of control	Difficulties in prioritizing work	Heavy workload	Insufficient team collaboration	Insufficient knowledge and training	Insufficient management support	Impact of work-related stress on the nurses’ private lives	Impact of the pandemic on nurses’ private lives

### Feelings of inadequacy and dissatisfaction contribute to work-related stress

The results indicated that the registered nurses experienced different types of work-related stress. The main theme “*Feelings of inadequacy and dissatisfaction contribute to work-related stress*” was divided into three categories: *Difficulty coping with their work tasks, Insufficient support, *and* Work-related stress affects private lives.*

### Difficulty coping with work tasks

Some of the difficulties connected to coping with their work were according to the registered nurses: *Lack of time, Understaffing, Lack of control, Difficulties in prioritizing work, Heavy workload*.

#### Lack of time

The participants described that they did not have enough time and resources to keep up with everything that should be done. Meanwhile, the number of patients was not decreasing, and there were always people in need of care. According to the participants, stress meant not having time to do the things they needed to do and sometimes they needed to be done quickly, depending on the situation. This problem was described as a new kind of stress that may not be there initially, but this was caused by a consistent lack of time.

*“I may have to be fast depending on what happens to the patient and then I might feel further stress that was not there earlier” (Interviewee n.6).*

#### Understaffing

The participants experienced too few staff in relation to the number of work duties and patients, which contributed to stress at work. They described the fact that they were understaffed, and it appeared that they were worried about not being able to do their work satisfactorily. Understaffing made them feel inadequate because they had more to do than they could handle. They described how the understaffing put tremendous pressure on them, leading to a more significant amount of stress.

*“We are very… very understaffed and at the same time we have new nurses who should get the right introduction, they should have the right support” (Interviewee n.3).*

#### Lack of control

The participants describe the lack of control as one of the causes which increased stress among them. The participants felt having no control over their work. They had no autonomy in the workplace, and this could likely lead to work-related stress. Nevertheless, there were times when the participants felt that they lost control, and not having control for them meant doing things more slowly, because they felt that the tasks needed more time or not having a plan B in the event of unexpected incidents.

*“Everything that you feel that you do not have control over can create stress” (Interviewee n.2).*

#### Difficulties in prioritizing work

Participants said they felt they had to prioritize in the workplace constantly as the workload increased and eventually, that could lead to decreased patient safety. Every time they had to prioritize, they experienced this as a stressful situation because they felt that they had not done enough for the patients and their relatives.

*“We are nurses we are not superhumans” (Interviewee n.9).*

The participants described how learning to prioritize goes beyond knowing how to use time properly. Making the wrong choice when prioritizing and planning can be problematic and be stressful. The participants said person-centered care should be prioritized to a greater extent to reduce stress in the workplace.

*“Learning to prioritize can reduce stress. To do one thing at a time” (Interviewee n.10).*

#### Heavy workload

The participants described how they had a large amount of work during a workday, which lead to increased stress. The results showed that participants who experienced a high workload and stress level at their workplace could have increased dissatisfaction with their work and either changed their place of work or left the profession, which increased the workload for those who remained.

*“Work-related stress is for me a large amount of work no matter what the work procedures or the mental pressure are” (Interviewee n.3).*

### Insufficient support

Insufficient support described how the participants experienced stress and what work-related stress meant to them. Their responses were divided into three subcategories: *Insufficient team collaboration, Insufficient knowledge and training, Insufficient management support*.

#### Insufficient team collaboration

The registered nurses felt that the physicians’ work in the care team was insufficient, and this could contribute to experiences of work-related stress. It also emerged that the participants experienced difficulties contacting a physician and getting a response. They needed to call the doctor several times so they could get advice about a particular patient. This situation was stressful because participants described cases when there was urgent to get in touch with a physician and they could not.

*“Then it can be stressful if you need a consultation from a doctor. You call them several times and no feedback. It can also be stressful” (Interviewee n.12).*

The participants described how support and cooperation between colleagues needed to be improved. They outlined the importance of competence and the cooperation between the various healthcare providers to work towards the same goal.

#### Insufficient knowledge and training

The participants revealed that working with patients made them doubt their professional nursing skills. Some of the participants mentioned that they wanted further education in their profession in order to be more proactive and feel confident in their work. The amount of work overload and the responsibility for other nursing staff with insufficient knowledge made them feel stressed, and the participants made clear that, this was a cause of work-related stress.

*“This uncertainty about their skills. I have no further education to lean on. I would like that. This too this is not going to be good. Even if you try” (Interviewee n.11).*

#### Insufficient management support

From the interviews, it emerged that the participants experienced a lack of support from the management, which contributed to an increased feeling of stress among them. According to the registered nurses, some of the managers did not have a degree in nursing, and they did not understand the nurse’s perspective or know what the role or the duties of the registered nurse. The participants described how it was important to have a manager who was on site, empathetic and was willing to find compromises with the registered nurses. According to the participants, this could help to reduce work-related stress. The participants said that a supportive management could lead to less stress being perceived as less among the registered nurses.

*“Cooperation with the manager is important” (Interviewee n.3).*

### Work-related stress affects private lives

In this category, participants described how the registered nurses experienced stress and how it affected their private life daily. In addition, they also talked about how their private life was affected in relation to the ongoing covid-19 pandemic. The two subcategories were: *Impact of workrelated stress on the nurses’ private lives* and *Impact of the pandemic on nurses’ private lives*.

#### Impact of workrelated stress on the nurses’ private lives

The participants reported feeling tired and sometimes irritated, and they described how this could also affect their quality of life. The participants described how working as a registered nurse was often stressful. They perceived that stress at work caused negative consequences for both physical and psychosocial well-being. Fatigue was experienced as a significant problem for the participants. It was noticeable during working hours, but it also affected the person in their spare time. The participants described how they felt fatigued, drained of energy, and they often did not remember things, which frightened them. They felt there was a lot of stress at work. For some of them, it felt as if it could be challenging to maintain a professional approach at work, and they tried to hide their feelings and to do the best for the patient or the relatives of the patient, but it was difficult to do. Meanwhile, they described how this mix of feelings also affected the quality of their own life.

*“I get tired, tired in my body, tired in my head, tired in general… I get gloomy” (Interviewee n.2).*

#### Impact of the pandemic on nurses’ private lives

During the period when the interviews were conducted, a situation arose that had not existed before, and which led the participants feeling more stress. The situation was Covid19, and it has changed how everyone works. During a pandemic situation, there was added stress. The participants had significant concerns about the risk of being exposed to the disease at work or even spreading it to loved ones, but they were also uncertain about the future in their workplace. It was a dilemma that created working-related stress among the registered nurses in municipal care. For the participants, this new stressful environment, and the constant stream of new guidelines every week during the outbreak of Covid19 led to high work-related stress for the registered nurses. As a result, the workload became heavier, and the amount of stress increased. During the interviews, they also revealed that many and constant changes to rules and recommendations made the healthcare providers feel even more stressed about not having control over their work.

*“It was a day when everything really exploded at once, I got a call related to the Covid19 epidemic and there was one person who was infected and then within a quarter of an hour there was another who was infected, and all hell broke loose at once and everything had to be done now” (Interviewee n.4).*

## Discussion

Experiences of work-related stress of registered nurses working in municipal aged care facilities can cause problems in the organizational and social work environment. The registered nurses described that stress occurred as they had “*Difficulties coping with their work task”* and they could not perform their duties. These findings are in line with White et al. [16] who pointed out that lack of time contributed to dissatisfaction at work, which also significantly increases the risk of threats to patient safety and quality of care. The registered nurses in this study described that understaffing forced them to more duties than they could handle. The risk of decreased quality of care because of understaffing in nursing was also brought up by Semachew et al.[20] and entails inevitable, negative consequences, especially for the quality of care and work satisfaction among registered nurses.

The registered nurses in this study described the difficulties of prioritizing what needed to be done the most at work because they had difficulties coping with so many work tasks. They described feeling a lack of control because of the lack of time and heavy workload caused by lack of support from the management, which leads to increased stress. The job demand-control-support model by Karasek and Theorell [19] identifies the conditions at work that predispose employees to work-related stress. The nurses in this study stated that it was essential to have an empathetic manager present who could understand the registered nurses. They also reported how the quantity of their work led to increased stress. According to Karasek and Theorell [19], however, it is not always excessive workload that can cause harmful stress or risk of illness. A lack of knowledge and poor communication can have the same effect. The registered nurses in these interviews described a lack of communication and support from their managers in the nursing facilities and a lack of company training programs. They also expressed a need for support from the nursing management. These concerns also feature in Karasek and Theorell’s job demand-control-support model. According to Karasek and Theorell [19], support mechanisms are fundamental when trying to manage job-related stress, and social support means support from colleagues, management, family, and friends. The main idea of the model is that demand for work, control over work processes, and social support within the workplace all relate to the individual’s well-being. To combat conditions that can lead to physical and psychological illness is necessary. The authors emphasize the importance of social networks in reducing work-related stress and how they can help to reduce the risk of illness in the workplace.

The registered nurses in our study experienced that the communication in the team suffered when the registered nurses experienced work-related stress and that it harmed the patient care. The job demand-control-support model by Karasek and Theorell [19] provides that in a culture that prevents work-related stress, it is essential to have social support to fulfill the work tasks. Dagget et al. [23] also described that not enough support from management could lead to difficulties in workers doing their job correctly and eventually increase the work-related stress among the health care providers. The registered nurses in our study who experienced a heavy workload and stress were considering quitting their job. These findings have similarities with previous studies by Carlesi et al. [14] and Chiang et al. [15], who showed that registered nurses left their jobs due to work-related stress and work-related fatigue and because of the inferior quality of patient care. According to Karasek and Theorell [19], it is essential to have control over the work situation to feel good at work. The findings suggest that if nurses felt as if they had more support and understanding from management, their work-related stress may reduce. However, this seems challenging due to limited support from both management and colleagues.

The findings in this study showed that not having control over the work and communication problems were stressful. In concordance with the study of Josefsson (2012), the organization must function in a way that creates conditions for registered nurses to perform risk-free care for older people. Routines must be clear and unequivocal [6]. The stressful work situation described by the participants in this study contributes to sleeping difficulties. The tiredness could lead to a source of irritation and could also negatively affect the quality of life of the nurses and risk patient safety. Fatigue and sleeping disorders describe as problematic. Previous research provides work-related problems from stress such as neck disorders, sleep disorders, headache, and fatigue and how they affect the quality of life of registered nurses [6]. Nowrouzi et al. [13] argue that by improving employees’ health-related quality of life without stress, intervention programs must incorporate each ward’s context. These programs should promote social support, sleep quality, exercise, and managing smoking habits. The registered nurses experienced more work-related stress than usual during the interviews due to the Covid‑19 pandemic. According to the participants, Covid‑19 has changed how they work in some situations and has led to more stress. The primary concerns were the risk of being exposed to the disease at work and managing various incidents at the workplace. The pandemic led to frustration and feelings of not doing the work satisfactorily. Antonovsky [18] argues that what is essential for an individual’s mental health is dealing with reactions to stress. These stress responses can be positive, keeping people alert to danger, motivated, or adaptable to new situations. Stress is not an illness itself, but when experienced frequently, it can increase the risk of mental health conditions such as depression, anxiety, and various addictions. Antonovsky [18] shows that by thinking from a salutogenic perspective, the individual can focus on the positive and learn to deal with stress differently.

## Strength and limitations

As described by Polit and Beck [21] the trustworthiness of studies with a qualitative design can be debated, in terms of their dependability, conformability, credibility and transferability. The study was based on a qualitative approach and was performed using individual interviews with the participants, who were selected with a convenience sample. It may be a weakness because they work in organizations that may have similar working conditions that may limit the variation in the content of the interviews. However, the participants were very variable in age, sex, education and time they had worked as registered nurses. They worked at six municipal aged care facilities in a major city. Therefore, they should be an adequate variation which is reflected in the data and could be transferable to other similar care facilities. A strength of the study was that the used method was chosen because the authors were interested in investigating subjective experiences of work-related stress in the care of older people in municipal aged care facilities. The choice of method is supported by Polit and Beck [21] who describe that qualitative method is suitable for studying people’s experiences, behaviors, and feelings therefore this method was chosen for this study. The reason why a semi-structured interview was chosen instead of, for example, a structured interview was to get a deeper insight into the phenomenon and ensure that all topics were covered. The study’s credibility refers to the extent to which research is trustworthy in the data collection and analysis. To prove this, each step in the method was described, and examples of how the analysis was performed were illustrated in the method section. Before starting the interviews, all the interviewed persons received information regarding the purpose of the study, the approach, and how confidentiality would be managed and that they could discontinue their participation at any time if they wished. This information was given both in writing and orally [24]. A strength of the study was that it included seven women and five men, which is representative of this type of research study. The registered nurses who participated had a good age distribution and different lengths of work experience within municipal care and inpatient care. This could also be seen as a strength in the study. Regarding the dependability of the study, the focus has been on the participants’ experiences of work-related stress. The data collection was done through the help of an interview guide, and the interviews were recorded by the authors with a digital voice recorder. The interviews were done in Swedish and then translated into English. Since English is not the author’s native language, this could be a limitation for the study. Answers that were unclear or could be interpreted in several ways were listened to a few times to get a clearer understanding of their statements.

According to Polit & Beck [21] dependability is about the fact that the study can be replicated with similar informants, with similar conditions, and get similar results. In qualitative research, it is also important to discuss confirmability during the study. The authors have been faithful and transparent that all the data obtained during the interviews are original and no data has been modified to change meaning. However, in qualitative studies, it is difficult to completely achieve confirmability as a researcher. However, striving for confirmability should be the ambition for all authors in qualitative research [22].

This manuscript was written during a pandemic outbreak. During the interviews, the registered nurses described the Covid-19 pandemic as a situation that added more stress to their daily work, which changed the way of working.

## Conclusion

In this study, experiences of work-related stress among a number of registered nurses were described and factors contributing to stress identified. Better collaboration in the care team and having an understanding manager is of great importance for reducing work-related stress. When the registered nurses who experienced stress received support to help deal with their situation, better conditions were created in order to provide a meaningful everyday life for the individual. With more of the world’s population suffering from work-related stress conditions, we must all be more proactive in reducing work-related stress.

The study can provide a basis for further research about work-related stress experienced by registered nurses in the care of older people at municipal aged care facilities. Future interventions should focus on introducing new and effective strategies for managing stress in the workplace and if possible, on a larger scale.

## Data Availability

The datasets used and/or analysed during the current study are available from the corresponding author on reasonable request.

## Abbreviations

WHO: The World Health Organization.
